# Gut microbiota: a promising new target in immune tolerance

**DOI:** 10.3389/fimmu.2025.1607388

**Published:** 2025-09-18

**Authors:** Nourah Almansour, Fatema Al-Rashed, Khubaib Choudhry, Hend Alqaderi, Sardar Sindhu, Fahd Al-Mulla, Rasheed Ahmad

**Affiliations:** ^1^ Department of Immunology & Microbiology, Dasman Diabetes Institute, Dasman, Kuwait; ^2^ Department of Human Biology, University of Toronto, Toronto, ON, Canada; ^3^ Department of Public Health and Community Service, Tufts University School of Dental Medicine, Boston, MA, United States; ^4^ Animal & Imaging Core Facilities, Dasman Diabetes Institute, Dasman, Kuwait; ^5^ Department of Translational Research, Dasman Diabetes Institute, Dasman, Kuwait

**Keywords:** gut microbiota, immune tolerance, immune modulation, Treg, probiotics

## Abstract

Gut microbiota research has highlighted its pivotal role in human health and disease. Its composition is shaped by diet, genetics, age, and environmental factors. When the balance of these microbes is disrupted (dysbiosis), it can contribute to health problems like metabolic, inflammatory, and mental disorders. The microbiota supports digestion, fermentation, and vitamin production, which are essential for overall health. The gut microbiota has emerged as a critical modulator of immune function, with increasing evidence highlighting its role in establishing and maintaining immune tolerance. Despite significant advances in understanding the interactions between the gut microbiome and immune system, gaps remain in the literature regarding the specific mechanisms through which microbiota influences immune tolerance. This review aims to address these knowledge gaps by synthesizing current research on the microbiota impact on immune tolerance, emphasizing key factors such as microbial diversity, metabolic byproducts, and the microbiota interaction with immune cells, specifically focusing on the role of microbial tryptophan metabolites in PD-1/PD-L1 tolerance. We also highlight critical areas for future research, including the identification of microbial species or strains that can modulate immune tolerance, the influence of diet and environmental factors on microbiota composition, and the development of microbiota-based therapies. By bridging these gaps, this review seeks to provide a comprehensive understanding of the mechanistic role of microbiota immune tolerance and its potential as a novel therapeutic target for autoimmune and inflammatory diseases.

## Introduction

### An overview of gut microbiota and its function

The exploration of gut microbiota has undergone remarkable progress over the past few decades. Initially, the presence of microorganisms in the gut was acknowledged in the late 19th and early 20th centuries, but their roles were not largely unraveled (1). It was not until the late 20th century, with the beginning of advanced molecular techniques such as 16S rRNA gene sequencing, that researchers began to uncover the vast diversity and complexity of the gut microbiome. The Human Microbiome Project, launched in 2007, marked a significant milestone, mapping the resident microbial communities in different parts of the body and illuminating the profound impact these microorganisms could have had on human health (2).

The gut microbiota consists of a diverse community of microorganisms, including bacteria, viruses, fungi, protozoa, and archaea (3) with bacteria being the most extensively studied microbiome component (4). These microbes colonize the gastrointestinal tract, with the colon harboring the densest populations. The composition of the gut microbiota is highly individualized and influenced by diverse factors such as diet, genetics, age, and environment (5). Healthy gut microbiota is characterized by a balanced diversity of species which is essential for maintaining body homeostasis. Disruptions in this balance, known as dysbiosis, have been increasingly linked to a wide range of morbid conditions including obesity, diabetes, inflammatory bowel disease (IBD), and even neuropsychiatric disorders (6, 7).

Functionally, gut microbiota plays a central role in numerous physiological processes. It is instrumental in the digestion and fermentation of complex carbohydrates, leading to the production of short-chain fatty acids (SCFAs) like acetate, propionate, and butyrate (8). These SCFAs not only serve as an energy source for the host but also have anti-inflammatory properties and contribute to the maintenance of the gut barrier (9). Additionally, the gut microbiota is involved in the synthesis of essential vitamins, such as vitamin K and certain B vitamins (10, 11), and in the metabolism of bile acids and other dietary components (12). These metabolic activities are crucial for nutrition, immune function, and maintaining overall health of the host. Beyond metabolic functions, the gut microbiota plays a pivotal role in immune system modulation. It aids in the development and maturation of the immune system, particularly in early life, and helps in maintaining a balanced immune response throughout life (13, 14). The microbiota also protects against pathogenic infections by outcompeting harmful microbes and producing antimicrobial substances (15). Emerging research has also highlighted the gut-brain axis, a complex communication network linking the gut microbiota with the central nervous system. This axis influences the brain function and behavior, and dysbiosis has been associated with mental health disorders such as depression and anxiety (16). As research continues to unfold, the gut microbiota is increasingly recognized as a key player in human health, with potential therapeutic implications for a variety of diseases.

## Role of gut microbiota in immune modulation

This diverse community of microorganisms can impact numerous health and disease-related factors, given the extensive genetic repertoire of genes carried by the various bacterial species present in the gut. The gut microbiota plays a crucial role in immune modulation, influencing both local and systemic immune responses. Studies have revealed that the gut microbiota can affect multiple functions in the human body, including the immune system through factors that affect its modulation (altered responses to maintain immune balance), maturation, and functioning (17). In this regard, gut microbiota help educate and train the immune system to distinguish between harmless substances (foods and commensals) and harmful invaders (pathogens) (18). Not surprisingly, germ-free animals have been found to have underdeveloped immune systems (19).

**Figure 1:**
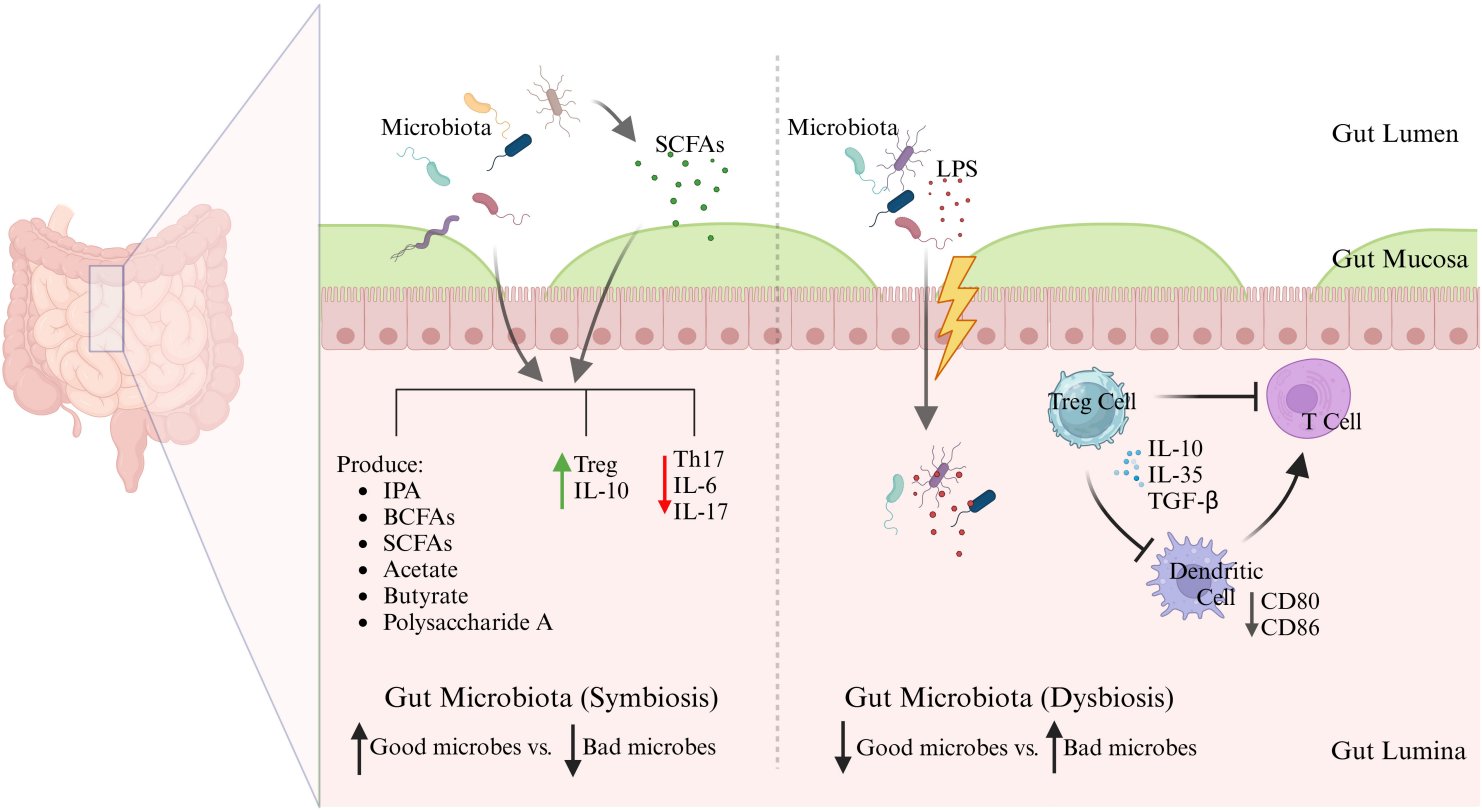
Symbiosis vs dysbiosis in gut microbiota. Schematic illustration demonstrates the immunological consequences of a balanced (symbiotic) versus disrupted (dysbiotic) gut microbiota. In symbiosis, commensal microbes produce SCFAs and other beneficial metabolites that reinforce barrier integrity and promote anti-inflammatory Treg responses via IL-10. In contrast, dysbiosis favors LPS-producing pathobionts, promoting barrier disruption, dendritic cell activation, Th17 skewing, and systemic inflammation through TLR4 signaling. These microbial shifts influence not only local gut immunity but also systemic inflammatory tone, linking gut health to chronic diseases.

Gut microbiota is critical to shaping the immune system during infancy. It contributes to the training of the immune system, enabling it to identify harmful pathogens while recognizing harmless substances, thus facilitating immune tolerance and preventing excessive immune reactivities to non-threatening antigens (20). Numerous studies have indicated that the maturation and function responses of major immune effector cells, including T cells, B cells, and dendritic cells, are significantly influenced by the microbiota (18, 21). Besides, gut microbiota contributes to the maturation of gut-associated lymphoid tissue (GALT) (22). A healthy microbiota produce beneficial metabolites, maintains the integrity of the intestinal epithelial barrier, preventing the translocation of pathogenic microbes and toxins from the gut lumen into the bloodstream which could lead to the activation of inflammatory responses (23, 24).Recent scientific research has elucidated the complex interaction between gut immune modulation and lipopolysaccharides (LPS), as illustrated in **Figure 1**. LPS are potent endotoxins derived from the outer membrane of Gram-negative bacteria and are recognized by the immune system as signals of bacterial invasion, leading to the activation of toll-like receptor 4 (TLR4) and subsequent downstream inflammatory cascades. Chronic exposure to LPS, often through a compromised gut barrier or dysbiosis, has been linked to systemic inflammation, contributing to conditions such as metabolic syndrome, insulin resistance, and even neuroinflammation (25). Recent studies emphasize the role of gut microbiota in modulating immune responses to LPS. For instance, certain beneficial bacteria produce SCFAs like butyrate, which have been shown to strengthen the gut barrier and reduce LPS translocation into the bloodstream (26). Furthermore, dietary interventions including high-fiber diets and specific probiotics, have demonstrated potential in reshaping gut microbiota to lower LPS levels and mitigate its inflammatory effects. Accumulating evidence underscores the importance of targeting the gut microbiome as a potential strategy to manage and prevent chronic inflammatory diseases associated with LPS. A recent study demonstrated that a high-fiber diet can reshape gut microbiota by preferentially supporting the beneficial bacteria, such as *Lactobacillus*, *Bifidobacterium*, and *Akkermansia*, while suppressing the harmful bacteria like *Desulfovibrio*and *Klebsiella*. This selective modulation in gut microbiota composition was associated with reduced serum LPS levels, in **Table 1**, which in turn mitigated inflammation and improved emotional mood in patients with type 2 diabetes (T2D) (37). Additionally, another interesting study explored the impact of a fermented-food diet compared to a high-fiber diet. It was found that while a high-fiber diet had limited effects on gut microbiota diversity, fermented foods significantly enhanced microbiome diversity and reduced inflammatory markers, providing further evidence of dietary influence on gut health and immune modulation (38).

**Table 1:** Gut bacteria and their products and functions.

Bacteria	Products	Functions	Adaptive immune functions	Human vs. murine evidence	Clinical correlations	References
*Lactobacillus plantarum*	Anti-inflammatory cytokines	Enhances intestinal barrier repair, modulates immune responses, reduces IL-6 and IL-17 levels, and increases IL-10 levels	Reduces inflammation and boosts IL-10. Regulates cytokine levels and reshapes the gut microbiome.	Both; murine restoration of DC function	Restores DC tolerogenicity; age-related immune decline	(27, 28)
*Akkermansia muciniphila*	Anti-inflammatory mediators	Strengthens the intestinal barrier, reduces inflammation, and balances cytokine levels	Promotes anti-inflammatory environments. Lowers IL-6 and IL-17 levels while boosting IL-10 production. Repairs the intestinal barrier.	Both; enriched in lean/healthy human subjects	Inverse correlation with obesity and inflammation	(27)
*Clostridium*sp*orogenes*	Indole-3-propionic acid (IPA), branched-chain fatty acids (BCFAs), short-chain fatty acids (SCFAs)	Promotes immune balance by reducing inflammation, suppressing T-helper 17 (Th17) cells, and enhancing Foxp3+ regulatory T cells (Tregs)	Produces metabolites like SCFAs and IPA. Enhances Treg activity, suppresses Th17 cells, and protects against colonic inflammation.	Primarily murine; colitis protection in mice	Protective effect against colitis in models	(29)
*Bacteroides fragilis*	Polysaccharide A	Induces Treg development, supporting immune tolerance	Produces polysaccharide A, which induces Treg cell development. Shapes immune responses toward tolerance.	Both; PSA effects shown in mice and human DCs	Reduced in IBD patients; Treg support in trials	(30)
*Segmented Filamentous Bacteria (SFB)*	Pro-inflammatory mediators (e.g., IL-17 stimulators)	Modulates Th17 and Treg balance, supports mucosal immunity, regulates cytokine production	Promotes the Th17 cells differentiation critical for mucosal immunity. Maintains the balance between Th17 and Treg cells.	Murine; key for Th17 in mice only	Not present in humans; no clinical utility	(31–33)
*Faecalibacterium prausnitzii*	Short-chain fatty acids e.g. butyrate	Supports gut barrier integrity, exerts anti-inflammatory effects, and promotes Treg differentiation	Produces butyrate, which supports Treg development. Exhibits anti-inflammatory properties.	Primarily murine; human association limited	Reduced in T2D, IBD; correlates with low butyrate	(34)
*Eubacterium rectale*	Butyrate	Enhances Treg activity and maintains intestinal health	Contributes to immune tolerance and gut homeostasis by maintaining barrier function and Treg differentiation	Primarily murine; inferred from SCFA levels	Correlates with healthy microbiota in IBD meta-analyses	(34)
*Bifidobacterium*spp.	Acetate and other SCFAs	Maintains gut barrier integrity, supports growth of beneficial microbes, modulates immune responses	Produces SCFAs like acetate, contributing to immune homeostasis. Modulates T cell responses and maintains gut epithelial integrity.	Human meta-analyses and murine models	Higher in infants with better gut health; supports immunity	(34)
*Desulfovibrio*	Hydrogen sulfide	Potentially disrupts gut homeostasis; reduction correlates with decreased inflammation	High levels are associated with dysbiosis and increased inflammatory responses. May indirectly influence adaptive immunity through modulation of inflammatory pathways.	Limited human data; murine inflammatory profiles	Elevated in IBD patients; potential marker of dysbiosis	(25)
*Klebsiella*spp.	Lipopolysaccharides (LPS)	Triggers inflammation: reduction improves immune regulation	Overgrowth is linked to gut dysbiosis and pro-inflammatory cytokine release. Contributes to adaptive immune dysregulation in inflammatory conditions.	Both; increased in human dysbiosis and mice	Overgrowth seen in metabolic and inflammatory diseases	(25)
*Helicobacter*spp.	Pathogenic antigens	Disrupts immune balance, leading to pathogenic Th17 expansion unless regulated	In certain contexts, influences Treg development via specific antigen-presenting cells (APCs) like RORγt+ APCs. Dysregulation can lead to pro-inflammatory Th17 cell expansion.	Murine gut studies; human cancer links weak	Linked with gastric cancer and dysbiosis	(35)
*Fusobacterium nucleatum*	ADP-heptose (pro-inflammatory metabolite)	Promotes inflammatory cytokines (IL-6, TNF-α), contributes to colorectal cancer progression	Produces pro-inflammatory cytokines like IL-6 and TNF-α in specific conditions (e.g. colorectal cancer). Creates a pro-inflammatory microenvironment but may also impact adaptive immunity in disease states.	Human colorectal cancer tissue; murine mechanistic	Associated with CRC, pro-inflammatory gene expression	(36)

There is also evidence that components of gut microbiota can interfere with TLR4 recognition by LPS, dampening the inflammatory immune response. A study showed that total LPS from the human gut microbiome was non-immunogenic and could actively inhibit TLR4-dependent cytokine production (27). This study found that underacylated forms of LPS, such as those produced by *Bacteroidales*bacteria, silenced TLR4 signaling across the gut microbiota consortium (27). Mechanistically, the lipooligosaccharide produced by *Akkermansiamuciniphila*lacks the O-antigen and exhibits structural modifications (monophosphorylation and acetylated sugars), which interfere with TLR4 binding and rather shift to anti-inflammatory TLR2 signaling (39). Also, certain lactic acid bacteria have been shown, *in vitro*, to suppress the expression of pro-inflammatory cytokines, including TNF-α and IL-6. The key mechanisms involved upregulation of the negative regulators of TLR4 signaling (e.g., Tollip, IRAKM, and SIGIRR) and suppression of downstream NF-κB and MAPKs pathways (27). A study in humans demonstrated that actual LPS-induced immune responses and gut microbiome composition did not predict the initial LPS response or the development of endotoxin tolerance *in vivo* (40).

However, it is also important to caution that high-fiber diets, while generally found to be beneficial, can worsen a functional gastrointestinal (GI) disorder, such as irritable bowel syndrome (IBS), and lead to microbiome-related complications in some individuals due to interactions between fiber and the gut microbiota, immune system, and intestinal environment (41). Many dietary fibers, especially fermentable ones, are broken down by gut microbes into SCFAs and produce gas. This is because some dietary fibers (such as inulin, fructooligosaccharides, and galactooligosaccharides) contain FODMAPS, i.e., Fermentable Oligosaccharides (fructans, galacto-oligosaccharides), Disaccharides (lactose), Monosaccharides (fructose), and Polyols (mannitol, sorbitol), which are readily fermentable but poorly absorbed carbohydrates and, in some (intolerant) people, can cause gastric sensitivity symptoms such as gas, bloating, abdominal pain, diarrhea, and constipation (42). In IBS, such diets may cause mucosal stress, worsen visceral hypersensitivity, intensify symptoms, and lead to microbial imbalance or dysbiosis.

**Figure 2:**
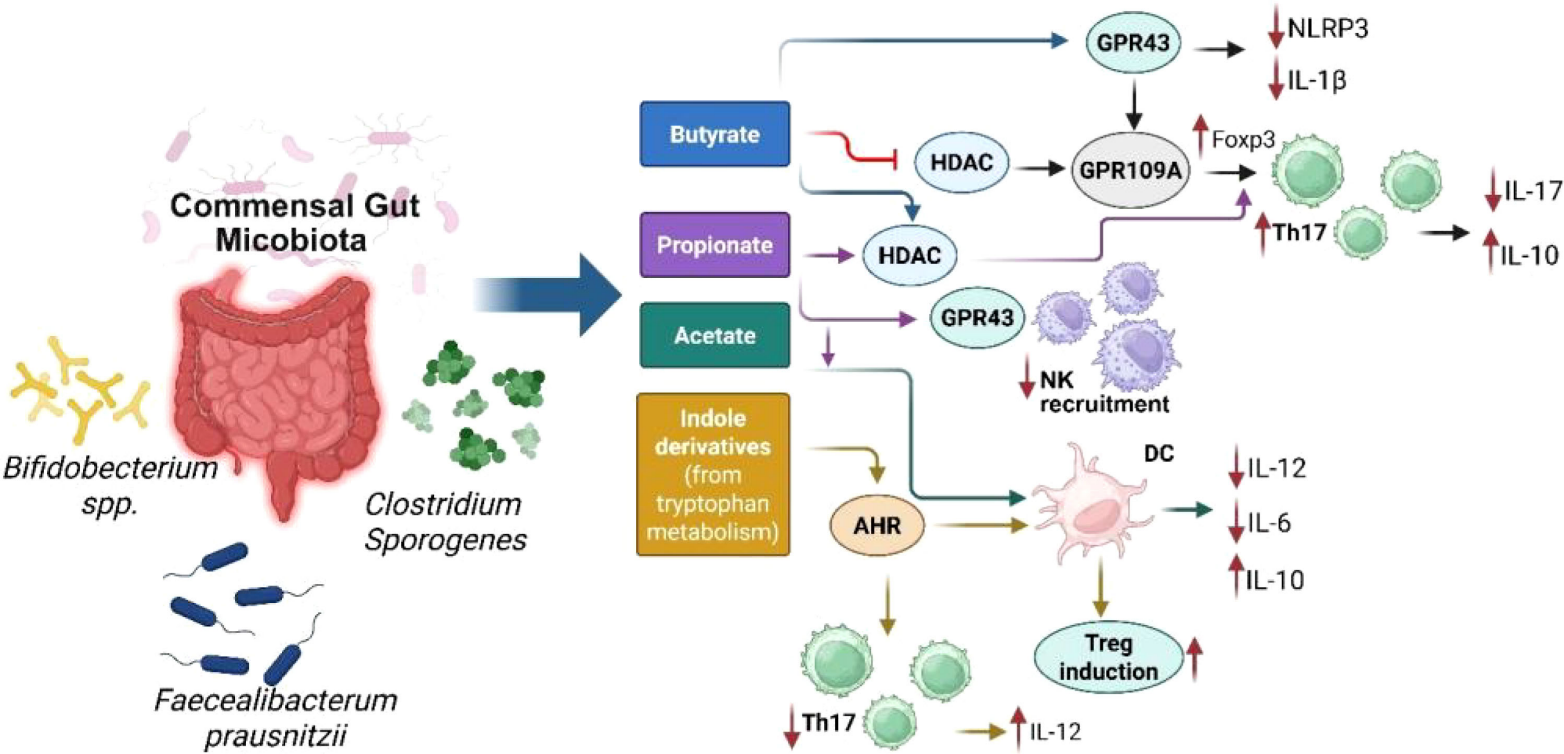
Schematic representation of key microbial metabolites (short-chain fatty acids and indole derivatives) produced by commensal gut microbiota (e.g., *Bifidobacterium*spp., *Clostridium*sp*orogenes*, *Faecalibacterium prausnitzii*), and their receptor-mediated effects on host immune function. SCFAs (butyrate, propionate, acetate) interact with receptors such as GPR43 and GPR109A or inhibit histone deacetylases (HDAC), leading to NLRP3 suppression, FoxP3+ Treg induction, and decreased IL-17/IL-1β secretion. Indole derivatives from tryptophan metabolism activate AHR (aryl hydrocarbon receptor), promoting anti-inflammatory pathways and Treg-supporting dendritic cell (DC) phenotypes. Collectively, these metabolites orchestrate immune homeostasis through downregulation of pro-inflammatory cytokines (e.g., IL-6, IL-12) and enhancement of regulatory networks (e.g., IL-10).

It is noteworthy that gut microbiotas have the capacity to stimulate the activation of interleukin 1β (IL-1β) and IL-17 through the action of commensal microorganisms, utilizing the TLR-MyD88 signaling pathway (43). Recent research has revealed that metabolites from the intestinal microbiota, particularly SCFAs and bile acids, are significant in the regulation of regulatory T cells (Tregs) (44). The microorganisms, such as *Akkermansia muciniphila* and *Lactobacillus plantarum*, mentioned in **Table 1**, are symbiotic bacteria which are instrumental in creating an anti-inflammatory environment by lowering the levels of IL-6 and IL-17, while boosting IL-10 levels in the bloodstream. These microbiotas exert this anti-inflammatory effect by regulating cytokine levels, repairing the intestinal barrier, and altering the gut microbiome composition to various degrees (45). The change in microbiota from newborn to adult stages boosts neutrophil production in the bone marrow. This happens since the microbiota stimulates intestinal IL-17A/IL-23, which raises the levels of granulocyte colony-stimulating factor (G-CSF) in the blood, encouraging neutrophil generation, and the process is called neutropoiesis. Mechanistically, gut microbes ferment dietary fiber into SCFAs, such as butyrate, propionate, and acetate, which bind to G-protein coupled receptors (GPCRs) like GPR43 on bone marrow stromal and immune cells. GPR43 activation leads to granulopoiesis, including neutropoiesis (**Figure 2**), (46). Once these neutrophils are released into the bloodstream, the microbiota still plays a potential role by enhancing the survival and function of neutrophils through SCFAs. Interestingly, in this regard, germ-free mice exhibited diminished neutrophil counts and impaired immune responses, which were reversed by supplementation of SCFAs (47, 48). Among other mechanisms, gut bacterial components, such as LPS, peptidoglycan, and flagellin, translocate through the gut mucosa and stimulate TLR4/2 signaling on hematopoietic and stromal cells in the bone marrow, upregulating G-CSF expression that drives proliferation and differentiation of neutrophils. In this regard, germ-free or antibiotic-treated mice lacked increased neutrophil production following tonic TLR stimulation (49). Gut microbiota also regulates the expression of TNF-α and IL-1β, which are involved in granulopoiesis during infection or inflammation (**Figure 2**) (46). Continued exposure to gut microbial products induces a functional reprogramming of myeloid progenitors in the bone marrow, called “trained immunity”. Such cues result in faster neutrophil responses to infections. Oral administration of gut bacterial-derived β-glucans was found to prime bone marrow progenitors for increased neutrophil production and responsiveness (35). Without the supportive role of microbiota, circulating neutrophils struggle to enter tissues during inflammation, and their ability to effectively fight invading pathogens is compromised (29, 50). Thus, the microbiota-bone marrow axis is an emerging target for controlling inflammatory diseases, infections, and cancer.

The SCFAs derived from microbiota reduce the inflammatory response of intestinal macrophages while simultaneously boosting their antimicrobial capabilities (30). The microbiota influences the balance between pro- and anti-inflammatory responses. SCFAs exert anti-inflammatory effects and help in maintaining immune homeostasis (51). Emerging evidence suggests that the gut microbiota plays a role in influencing neuroimmune interactions through the microbiota-gut-brain axis, potentially modulating central nervous system function and contributing to mood disorders (52).

## The gut Role of gut microbiota in immune tolerance

microbiota plays a critical role in the establishment and maintenance of immune response by influencing various immune cells and pathways. One of the critical functions it performs is promoting immune tolerance, a process that prevents harmful immune reactions against the body’s own tissues or harmless external antigens. Through interactions with immune cells and modulation of key signaling molecules like cytokines and chemokines, the gut microbiota helps maintain a balance between immune defense and tolerance, ensuring overall immune homeostasis (31).

### Introduction to immune tolerance

Immune tolerance refers to the ability of the immune system to distinguish between the body’s own cells and beneficial microbes, avoiding attacks on them while still effectively responding to harmful pathogens. This delicate equilibrium is essential for maintaining immune homeostasis and preventing immune responses from mistakenly targeting self-antigens or non-harmful commensal organisms (32). The process of developing immune tolerance starts in early life, involving mechanisms like central tolerance in the thymus, where self-reactive T cells are purged, and peripheral tolerance develops which regulates immune cells throughout the body tissues (33).

The importance of immune tolerance is evident in its ability to avert autoimmune diseases in which the immune system erroneously attacks the body’s own cells. If tolerance mechanisms are ineffective, the autoimmune diseases such as lupus, type 1 diabetes, and rheumatoid arthritis may develop (53). Additionally, immune tolerance supports a beneficial relationship with the commensal microbes in the gut, enabling the immune system to live in harmony with these microbes without inciting chronic inflammation (32). Immune tolerance is also essential for the success of organ transplants, as it helps prevent the graft rejection (54).

## Possible mechanisms underlying gut microbiota-mediated immune tolerance

Immune tolerance in the gut is vital for maintaining a balance between accepting beneficial commensal microbes and mounting a defense against the harmful pathogens. This intricate balance is maintained through various regulatory mechanisms that adjust immune responses accordingly. Gut microbiota are the key players in this system, as they help foster immune tolerance and prevent the immune system from mistakenly targeting harmless commensals or self-antigens.

### Regulation of Tregs

**Figure 3:**
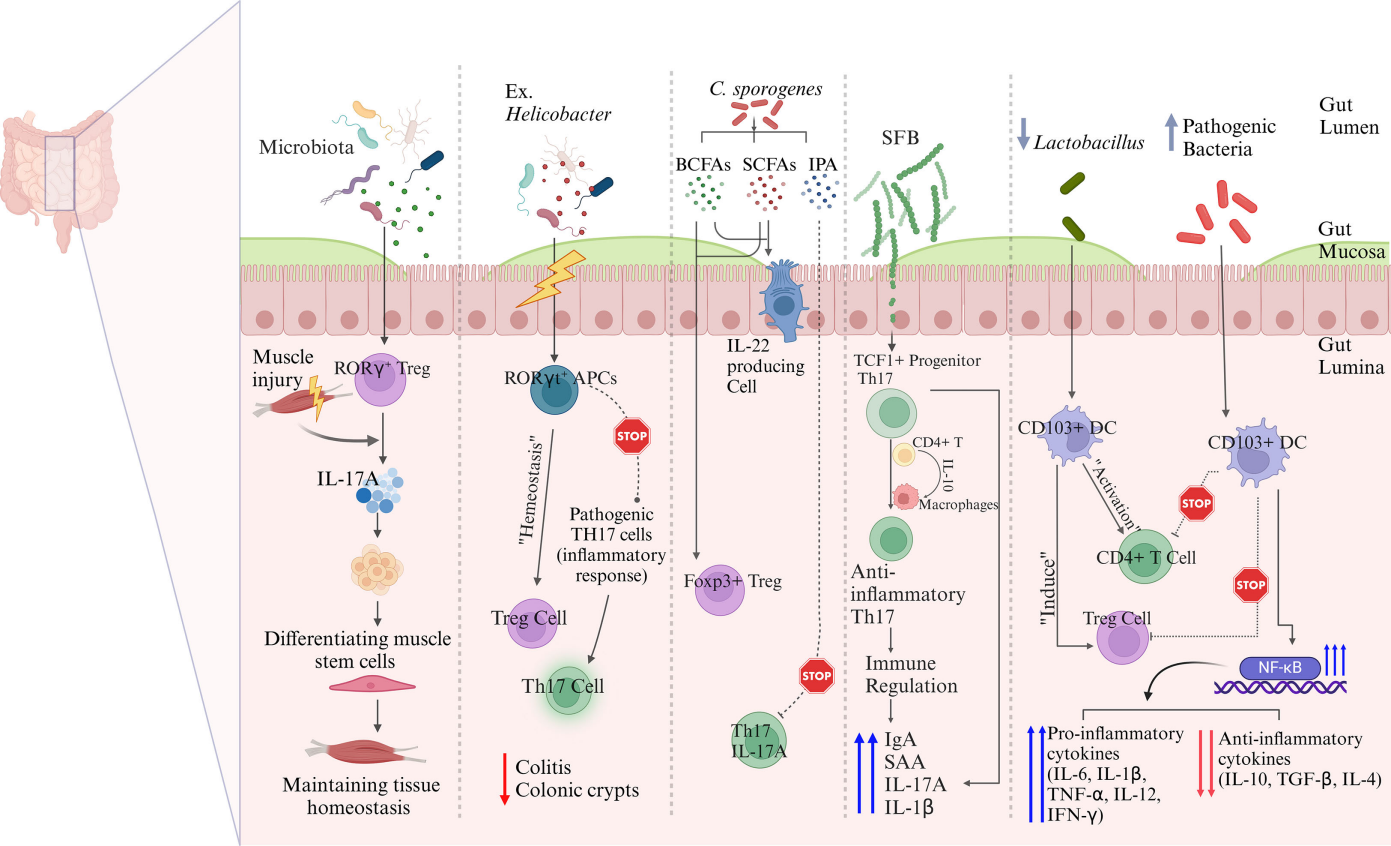
Possible mechanisms underlying gut microbiota-mediated immune regulations. Commensal microbiota influence immune regulation through multiple interconnected pathways. Microbiota-induced RORγt^+^ Tregs migrate to peripheral sites such as injured muscle, where they suppress IL-17A–driven inflammation and promote tissue regeneration. In the gut, RORγt^+^ antigen-presenting cells (APCs) facilitate the expansion of inducible Tregs while limiting the differentiation of pathogenic Th17 cells, thereby maintaining intestinal immune homeostasis. Microbial metabolites, including SCFAs and indole derivatives, promote the differentiation of Foxp3^+^ Tregs and restrain pro-inflammatory Th17 responses. Segmented filamentous bacteria (SFB) shape the development of anti-inflammatory Th17 cells from TCF1^+^ precursors via IL-10 signaling and macrophage crosstalk. CD103^+^ dendritic cells also contribute to immune tolerance by inducing Tregs and suppressing effector T cell activation through modulation of NF-κB signaling. These pathways collectively highlight the essential role of gut microbiota in balancing immune activation and tolerance across mucosal and systemic compartments.

Some gut bacteria are instrumental in the differentiation of Tregs which are essential for moderating excessive immune responses and enhancing tolerance to self and dietary antigens. Tregs play a major role in suppressing inflammatory immune responses. The presence of a balanced gut microbiota is instrumental in the activation of Tregs which produce anti-inflammatory cytokines, including IL-10 and TGF-β as well as additional cytokines, thus enhancing the process of immune tolerance. A recent study showed that gut-derived RORγ+ Tregs (depicted in **Figure 3**) influenced by the gut microbiota, accumulate in injured muscle where they regulate inflammation and support muscle repair. These cells help balance the stem cell proliferation and differentiation by shielding muscle stem cells from IL-17A, highlighting their broader role in maintaining tissue homeostasis beyond the gut (28). Kedmi et al. found that specific antigen-presenting cells (APCs) expressing the transcription factor RORγt, called RORγt+ APCs rather than classical dendritic cells, are crucial for promoting expansion of inducible T regulatory cells (iTregs) in response to gut bacteria like *Helicobacter* (**Table 1**, **Figure 3**). If these RORγt+ APCs or their associated factors are absent, pathogenic Th17 cells expand instead, disrupting the immune balance and promoting inflammation (55). Most Tregs are CD4+ and express the transcription factor FoxP3 which is critical for their function. Tregs suppress immune responses through various mechanisms, including direct cell contact, metabolic disruption, and the secretion of inhibitory cytokines (56). It was shown that the gut symbiotic bacterium *Clostridium*sp*orogenes* (**Table 1**, **Figure 1**) synthesizes metabolites including indole-3-propionic acid (IPA), branched-chain fatty acids (BCFAs), and SCFAs, all of which are essential for maintaining the immune balance within the intestine (57). In experiments involving mice colonized by *C.*sp*orogenes*, these metabolites indicated a reduction in inflammation through suppressing the Th17 cells and enhancing the activity of Foxp3+ Tregs and IL-22. This approach helps protect against colitis and prevents the deterioration of colonic crypts (**Figure 3**) (57).

Furthermore, specific symbiotic bacterial strains like *Bacteroides fragilis* (**Table 1**) produce polysaccharide A (**Figure 1**), which directly interacts with the immune system to induce Treg development. This demonstrates a sophisticated interaction between the gut microbiota and the host immune system where microbial metabolites and components actively shape immune responses toward tolerance (58).

The gut microbiota plays a significant role in regulating the activity of Th17 cells, which are involved in mucosal immunity and inflammation. Alongside Tregs, the balance between Th17 cells and Tregs is crucial for maintaining a state of immune tolerance and preventing the development of inflammatory diseases. Certain commensal bacteria, such as segmented filamentous bacteria (SFB) (**Table 1**, **Figures 2**, **3**), have been shown to promote the differentiation of Th17 cells in the gut. While Th17 cells are essential for protecting against pathogenic infections, their overactivation can result in excessive inflammation and contribute to autoimmune diseases (59). Therefore, gut microbiota helps maintain the delicate balance between Tregs and Th17 cells, ensuring that immune responses are appropriately modulated to prevent harmful inflammatory reactions (60). The Th17/Treg balance is remarkably influenced by gut microbiota, specifically through bacterial metabolites and host-microbe interactions at the mucosae (61). The bacterial communities and specific taxa that regulate the Th17/Treg axis for immune balance are discussed as follows. The bacteria that promote the Th17 differentiation (autoimmune and inflammatory responses) include the following: (i) Members of the *Enterobacteriaceae*family (e.g. *E. coli*), which produce Th17 in dysbiotic guts and are associated with Crohn’s disease and colitis (36, 62, 63); (ii) Segmented filamentous bacteria (SFB), which are often found in mice strongly induce Th17 expression in small intestine, via the mechanisms including adhesion to intestinal epithelial cells and induction of serum amyloid A that activates DCs for skewed induction of IL-6, IL-17, and IL-23 (64, 65); and (iii) *Prevotellacopri*, which is associated with promoted Th17 responses and intestinal inflammation, due to bacterial LPS production (66). This bacterium is also found enriched in certain autoimmune diseases, such as rheumatoid arthritis (67). The following bacteria are found to promote the Treg differentiation (leading to immune tolerance/homeostasis): (i) *Lactobacillus*species, such as *L. reuteri*and *L. plantarum*, which favor Treg differentiation by producing AhR ligands and tryptophan (Trp)/indole derivatives (68, 69).These bacterial metabolites activate anti-inflammatory pathways in the gut; (ii) *Clostridia*clusters IV and XIVa, including the genera *Ruminococcus, Faecalibacterium, Eubacterium*, and *Anaerostipes*. These bacteria produce SCFAs, such as butyrate, which induce colonic Foxp3+ Treg differentiation and immune tolerance via the mechanisms involving histone deacetylase (HDAC) inhibition and TGF-β signaling (**Figure 2**) (34, 69); and (iii) *Bacteroides fragilis*, which produces polysaccharide A, promoting the differentiation of Foxp3+ Tregs and IL-10 expression by stimulating TLR2 on DCs (70, 71). Of note, indole derivatives from commensal microbes, such as *Lactobacilli*and *Clostridia*, promote the Treg differentiation and IL-22 production via AhR-mediated signaling (69, 72). These derivatives also modulate Th17 responses, depending on the ligand type (73).

Brockmann et al. found that IL-10-producing commensal-specific SFB-responsive Th17 cells were derived from a TCF1+ progenitor population in the gut and acquired their anti-inflammatory phenotype within the small-intestinal lamina propria. IL-10 production by CD4+ T cells and IL-10 signaling in the intestinal macrophages were essential for maintaining IL-10 expression in Th17 cells. Functional assays further demonstrated that these Th17 cells suppressed effector T cell activity both *in vitro*and *in vivo*, underscoring their immunoregulatory capabilities in an IL-10- and c-MAF-dependent manner. This study highlights the pivotal role of Th17 cells in controlling immune responses and supporting mucosal homeostasis (74). A recent study showed that SFB significantly influences immune responses in the gut. Specifically, primary tissue slices from SFB-positive Taconic mice, when stimulated, showed up to a 15-fold increase in Th17 cell-associated mediators, such as serum amyloid A (SAA), and immunoglobulin A (IgA), compared to SFB-negative mice (75). Additionally, the distinct microbiome compositions between SFB-positive and -negative mice resulted in different frequencies of Th17 cells. Co-housing of mice demonstrated that the SFB-related immune phenotype could be transmitted. These results suggest that SFB played a crucial role in modulating immune responses (75). These findings were further corroborated by another study showing that SFB significantly enhanced the Th17 cell responses, leading to increased production of IL-1β (76).

### Role of ILC3s in microbiota-mediated immune tolerance

Beyond adaptive immune cells like Tregs and Th17, group 3 innate lymphoid cells (ILC3s) have emerged as pivotal regulators of intestinal immune tolerance. ILC3s are enriched in gut-associated lymphoid tissue and play a critical role in maintaining homeostasis at mucosal surfaces. They mediate tolerance to commensal bacteria and dietary antigens through antigen presentation, secretion of IL-22, and promotion of tolerogenic Treg responses (77). Notably, Lyu et al. demonstrated that ILC3s can selectively present antigens via MHC class II and drive the expansion of microbiota-specific Foxp3^+^ Tregs while simultaneously suppressing the differentiation of pro-inflammatory Th17 cells (78). This selective interaction fosters immune tolerance without compromising antimicrobial defenses. Furthermore, ILC3s interact closely with epithelial cells and DCs, contributing to epithelial barrier integrity and cytokine balance. Disruption in ILC3–Treg crosstalk has been linked to intestinal inflammation and IBD pathogenesis. Thus, ILC3s serve as a critical interface between the microbiota and the host immune system, fine-tuning the balance between tolerance and immunity.

### Antigen presentation

APCs are essential in priming and regulating immune tolerance to gut microbiota through a variety of mechanisms. Recent studies have underscored the importance of specific APC subsets in preventing inflammatory responses within the gut. Dendritic cells (DCs), macrophages, and B-cells all serve as APCs, with DCs being regarded as the most professional and specialized for this function (79). DCs are the key regulators of immune tolerance and inflammation in the gut, influencing T-cell differentiation to maintain gut homeostasis. Microbial products like SCFAs and polysaccharides can modulate DCs to promote a regulatory phenotype, favoring Treg induction and reducing pro-inflammatory responses (80). DCs produce anti-inflammatory cytokines, such as IL-10 and TGF-β, that promote the differentiation of Tregs. This process is crucial for maintaining immune tolerance to dietary antigens and commensal microbes. A recent study on how aging and gut dysbiosis affected DCs function (shown in **Figure 1**) reported that DCs from both aged and young mice with gut dysbiosis lost tolerance, failing to induce Tregs and control CD4+ T cell activation. The loss of tolerance in these DCs was linked to overactivation of NF-κB, increased levels of pro-inflammatory cytokines (IL-6, IL-1β, TNF-α, IL-12, IFN-γ), and a decline in anti-inflammatory cytokines (IL-10, TGF-β, IL-4), along with a reduction in *Lactobacillus*bacteria in the gut (81). Another study found that CD103+ DCs (shown in **Figure 3**), influenced by signals from epithelial cells, promoted non-inflammatory immune responses, while CX3CR1+ cells helped transfer antigens for tolerance. This study also explored the immunomodulatory effects of *Lactobacillus paracasei*CBA L74, highlighting the importance of nutrition and microbiota in infant health (82).

Dendritic cells type 2 (DC2s) play a significant role in regulating immune tolerance, specifically at mucosal and peripheral tissue interfaces. This subset (also called CD11b+ DCs) is involved in shaping T-cell responses, supporting a balance between tolerance and effector immunity (83, 84). DC2s are found in mucosal tissues (including gut, lung, skin), spleen, and lymph nodes. DC2s express CD11b+, CD11c+, MHC-II+, IRF4+, and SIRPα+ surface molecules, and mainly induce Th2, Th17, and Treg responses, depending on the context (85). DC2s regulate immune tolerance via the following key mechanisms: (i) Induction of Tregs: Mucosal DC2s induce Foxp3+ Tregs by upregulating the expression of immunoregulatory molecules (CD103, PD-L1, ICOS-L) and by increasing tolerogenic cytokines (IL-10, TGF-β, and retinoic acid) (86). These cues skew naïve CD4+ T-cells toward Treg lineage, supporting immune tolerance and mucosal homeostasis; (ii) Suppression of effector T-cells: In steady state or tolerogenic conditions, DC2s present antigens without involvement of co-stimulatory molecules, which leads to clonal deletion and T-cell anergy (87). DC2s continue inhibiting Th1 and Th17 responses in the absence of inflammatory signals. DC2s also favor IL-10 production by Tr1 cells in the peripheral tissues (88); (iii) Neuroimmune regulation: In lung and skin tissues, DC2s interact with neuronal and epithelial signals. In non-inflammatory conditions, these interactions support Treg differentiation, while in allergic conditions, DC2s favor Th2 responses (89); (iv) Microbiota-dependent programming: Gut and skin commensal microbiota prime DC2s to attain tolerogenic phenotypes (90). To this effect, microbial metabolites, such as SCFAs and indoles, interact with AhR on DC2s to promote IL-10 production. Thus, microbiota trains DC2s to induce tolerance toward harmless antigens related to commensals and foods; and (v) Differentiation into tolerogenic DCs (91, 92): Under cues, such as microbiota, TGF-β, and vitamin A, DC2s can transform into tolerogenic DCs, marked by the expression of indoleamine 2,3-dioxygenase (IDO) and β-8 integrin. Defects in DC2s have been related to autoimmunity, allergy, and oral hypersensitivity or intolerance to food antigens. Ngoie et al. investigated the role of migratory DC2s in mediating the mucosal Th17 response to gut commensal bacteria. DC2s, through their expression of CD40, Ccl17, and Ccl22, were found to be involved in the colocalization of Th17-polarizing cytokine IL-6, linking the cytokine environment to the functionality of these cells (93).

### Microbial influence on the expression of co-stimulatory molecules on APCs

Gut microbiota can profoundly influence the expression of co-stimulatory molecules on APCs, including DCs and macrophages – a key mechanism by which microbiota regulate immune tolerance vs. activation. The co-stimulatory molecules expressed on APCs are key signals required for T-cell activation, and their presence or absence determines whether T-cells will become activated effector T cells, remain anergic or non-responsive, or will differentiate into tolerogenic Tregs (58, 94, 95). These co-stimulatory molecules include the following: (i) CD80 (B7-1)/CD86 (B7-2): These co-stimulatory molecules bind to CD28 for T-cell activation or CTLA-4 for T-cell inhibition (96); (ii) PD-L1/PD-L2: These ligands bind to PD-1 for T-cell inhibition (97); (iii) Inducible co-stimulator ligand (ICOS-L): This ligand binds to ICOS on T-cells and this interaction promotes Treg differentiation (98); and (iv) CD40/CD70/OX40L: Binding of these co-stimulatory molecules promotes the differentiation of effector T-cells (99). Microbiota influences co-stimulatory molecular expression through the following mechanisms: (i) Tolerogenic co-stimulation induced by commensal microbiota: Microbes, such as *Lactobacilli*spp., *Bacteroides fragilis*, and *Clostridia*clusters IV/XIVa, can induce expression of PD-L1, ICOS-L, and suppress the expression of CD80/CD80 co-stimulatory molecules, promoting tolerogenic DCs and Treg induction (100). Polysaccharide A from *B fragilis*induces PD-L1 expression and TLR2-mediated IL-10 expression (101); (ii) Modulation of APC function by microbial metabolites (102): (a) SCFAs, such as butyrate, downmodulate CD80/CD86, upregulate PD-L1, inhibit HDACs in DCs, and induce tolerogenic DC phenotypes. (b) Trp metabolites, such as indole derivatives, induce AhR-mediated signaling to promote expression of IL-10 and PD-L1. These metabolites modulate APCs’ signaling to promote Treg differentiation. (c) Secondary bile acids inhibit the expression of pro-inflammatory co-stimulatory molecules, while increasing the expression of PD-L1 and IL-10-producing DCs; and (iii) Immunogenic co-stimulation induced by opportunistic microbes/pathobionts or in dysbiosis (103): Dysbiotic growths of pathobionts, such as *Enterobacteriaceae*members and *Prevotella copri*, or exposures to pathogen-associated molecular patterns (PAMPS) induce expression of CD40, CD80/CD86, and OX40L, which promotes Th1/Th17 polarization. These interactions induce inflammatory responses and autoimmune activation (103). *Segmented filamentous bacteria (SFB)*induced pro-inflammatory APC modulation in mice to promote the development of Th17 cells by co-stimulatory activation (104).

The microbiota-APCs interactions involve the following key pathways: (i) TLR2/TLR4 signaling (105): This interaction modulates the expression of CD80/CD86 co-stimulatory molecules and cytokine induction (based on context); (ii) AhR activation (as by Trp metabolites) (106): This interaction upregulates the expression of PD-L1 and IL-10, and suppresses inflammatory co-stimulation; (iii) HDAC inhibition (as by SCFAs) (107): This interaction promotes tolerogenic phenotypes via epigenetic silencing of pro-inflammatory gene expression; and (iv) NOD-like receptor (NLR) pathways (108): This interaction induces modulation of co-stimulation and altered cytokines’ expression (based on context). In conclusion, gut microbiota influences the expression of co-stimulatory molecules on APCs, which shapes the immune response toward immune tolerance or activation. This immune regulation is mediated by direct microbial interactions through TLRs and surface molecules, as well as by microbial metabolites, such as SCFAs, indoles, and secondary bile acids. Notably, a balanced microbiota promotes tolerogenic immune profiles, whereas dysbiosis or pathobionts can tip the immune balance toward inflammation and autoimmunity.

Regarding the question of whether the gut microbiota helps restore peripheral tolerance, central tolerance, or both, it is noteworthy that the gut microbiota orchestrates primarily the restoration and maintenance of peripheral tolerance (18, 102, 109); however, some evidence also suggests that it may indirectly influence central tolerance as well (110, 111). Gut microbiota plays a significant role in maintaining by: (i) Treg induction (109): Commensals, such as *Bacteroides fragilis*, *Lactobacillus*spp., and *Clostridia*clusters IV/XIVa, promote Foxp3+ Treg differentiation. In this regard, microbial metabolites like SCFAs (butyrate, propionate), Trp metabolism/indole derivatives, and secondary bile acids induce and stabilize tolerogenic APCs and Tregs in both the gut and peripheral circulation; (ii) Inhibition of proinflammatory T-cell subsets (21): A balanced or healthy microbiota suppresses Th1, Th17, and cytotoxic T lymphocytes (CTL) responses enhancing PD-L1 expression on APCs, AhR activation and by increasing expression of IL-10 and TGF-β; (iii) Maintaining barrier integrity (112): Gut microbes support epithelial integrity in the gut by upregulating expression of occludin/ZO-1, thus limiting translocation of antigens and inhibiting immune activation (113). Taken together, the gut microbiota actively induces peripheral immune tolerance, specifically in GALT, lungs, and skin. Regarding effects on central tolerance (114), while the thymus is not directly colonized by microbes, their indirect effects could be as follows: (i) SCFAs and Trp metabolites enter the circulation and can promote natural Treg development, thymic stromal cell development, and support thymic epithelial cell function; and (ii) Exposure to microbiota-associated antigens: In early life, exposure to microbial antigens related to commensals can help shape the thymic repertoire through the mechanism of systemic antigen presentation, driven by migratory DCs. This process contributes to a broad-scale central tolerance to gut microbes- and food-associated antigens and prevents autoimmunity in early life. Overall, gut microbiota is a key driver of peripheral immune tolerance via the induction of Tregs, anti-inflammatory signaling, and maintenance of gut barrier integrity. Nonetheless, gut microbiota can indirectly influence central tolerance, especially in early life, to maintain immune homeostasis and prevent autoimmunity.

The interactions between gut microbiota and macrophages significantly impact immune tolerance, resulting in the generation of immune signals and microbial metabolites that influence macrophage functionality. This dynamic interplay is vital for preserving immune homeostasis, as it helps to prevent auto-inflammatory and autoimmune diseases by encouraging a well-regulated immune response (115). Macrophages in the gut, influenced by microbial metabolites, tend to adopt a more anti-inflammatory, M2-like phenotype that supports tissue repair and immune tolerance. This contrasts with the pro-inflammatory M1 macrophages, which can drive inflammation when unchecked (116). Natural killer (NK) cells, crucial for detecting and responding to pathogens, are also modulated by the gut microbiota, which provides ligands for NK cell receptors, affecting their function and cytotoxicity (117). Recent advancements in the understanding of NK cell biology have led to the recognition of NK cells as a diverse subset within the innate lymphoid cell (ILC) family (118). An elegant study showed that the ILC3s and Tregs co-localized in the intestine-draining lymph nodes, played crucial roles as ILC3s promoted RORγt+ Tregs while suppressing Th17 cell expansion, thus helping maintain the immune balance (59). This study suggested that impaired ILC3-Treg interactions might contribute to inflammatory bowel disease (78).

The composition of the gut microbiota has a profound effect on cytokine production, serving as a fundamental mechanism for the maintenance of immune tolerance. Certain bacterial species stimulate the release of anti-inflammatory cytokines (IL-10 and TGF-β) which are critical in preventing overactivation of the immune system. These cytokines are instrumental in promoting Treg differentiation which is essential for achieving the immune homeostasis (119). In contrast, the pro-inflammatory cytokines, such as TNF-α and IL-1, which are necessary for defense against pathogens are tightly regulated by the microbiota to avoid chronic inflammation (120). Martin-Gallausiaux et al. found that *Fusobacterium nucleatum* (**Table 1**), stimulates the production of pro-inflammatory cytokines, such as IL-6 and TNF-α, in colorectal cancer cells through the release of ADP-heptose which contributes to an inflammatory microenvironment that supports tumor progression and enhances the survival of cancer cells (121). Recently, Haojie Ni et al. reported that the alkaloid sinomenine, derived from the root of the climbing plant Sinomenium acutum, regulated the cholinergic anti-inflammatory pathway which, in turn, inhibited the TLR4/NF-κB signaling pathway in scopolamine-induced Alzheimer’s disease mice. This regulation resulted in a reduction of pro-inflammatory cytokines (TNF-α and IL-1β) and an increase in IL-10 production (122). Thus, the modulation of these cytokines was shown to be closely linked to the maintenance of microbiota homeostasis (122). Another interesting study established a link between the gut microbiota composition and the expression of inflammatory markers in individuals with T2D. This study showed that the reduced microbial diversity and lower levels of beneficial bacteria were associated with increased levels of pro-inflammatory cytokines, such IL-6 and TNF-α, and this dysbiosis in gut microbiota led to systemic inflammation, indicating that the gut microbiota played a crucial role in upregulating inflammatory responses and contributed to the pathogenesis of T2D (123). Overall, balanced cytokine production ensures that the immune system remains tolerant to the harmless antigens while still defending against the pathogens. The gut dysbiosis can lead to reduced immune tolerance and disrupt the equilibrium of various immune cells including Tregs, Th17 cells, DCs, and macrophages. This disruption can compromise the intestinal barrier, leading to increased permeability, inflammation, and immune activation. In a sepsis model study, transferring the fecal microbiota from healthy male mice to recipient mice resulted in lower serum IL-1β concentrations, suggesting that a balanced gut microbiota could modulate immune responses and maintain immune tolerance (26). Overall, the interplay between gut microbiota and immune cells is vital for preventing inappropriate immune reactions and promoting a balanced immune system (124, 125). The various mechanisms underlying gut microbiota-mediated immune regulations are summarized below in **Figures 2**, **3**.

### Bacterial metabolites and immune modulation

The emerging studies have highlighted several potential mechanisms by which the gut microbiota contributes to immune tolerance - a crucial aspect of maintaining the immune system balance and preventing autoimmune diseases. One primary mechanism involves the modulation of Tregs, which play a vital role in maintaining immune tolerance (126). The gut microbiota influences Treg development and function through the production of metabolites such as SCFAs, which have been shown to enhance Treg activity and promote tolerance by suppressing inflammatory responses (127). Gut bacteria produce SCFAs, including butyrate, propionate, and acetate, through the fermentation of dietary fibers. These SCFAs promote the differentiation and expansion of Tregs in the gut environment, enhancing the immune tolerance (107). Certain colon bacteria like *Firmicutes*and *Bacteroidetes* (as mentioned in **Table 1**, **Figure 1**) metabolize complex carbohydrates into SCFAs. Butyrate is mainly produced by anaerobic bacteria (symbiotic bacteria) like *Faecalibacterium prausnitzii* and *Eubacterium rectale*, while acetate is more broadly produced by several species across different genera including the *Bifidobacterium* (128). Microbial metabolites play a crucial role in maintaining immune tolerance by regulating the activity of several immune effector cells including Tregs. These metabolites modulate the inflammatory responses to help prevent autoimmune reactions and maintain gut homeostasis. SCFAs play important roles in health, such as maintaining the integrity of the intestinal barrier, modulating immune responses, and exerting anti-inflammatory effects (129).

Lee et al. found that the SCFA supplementation in a murine model of colitis significantly reduced the gut inflammation and altered microbiota composition. This study showed that SCFAs might help in modulating immune responses and promoting gut health in inflammatory conditions (130). SCFAs, especially butyrate, enhance Treg development by activating the transcription factor FoxP3 which is crucial for Treg stability and suppressive function. Butyrate specifically increases histone acetylation in the FoxP3 promoter region, driving the expression of FoxP3 in naïve T cells. This regulatory mechanism has been highlighted in autoimmune disease models where butyrate enhances the Treg-mediated suppression of inflammation (131). Besides, McBride et al. reported that SCFAs epigenetically modulated CD4^+^T cells by reducing the production of pro-inflammatory cytokines, such as IL-17 and IFN-γ, while increasing the levels of anti-inflammatory IL-10. This study indicated that the SCFAs could influence immune regulation by altering the epigenetic landscape of inflammatory T cells, suggesting their potential therapeutic applications for inflammatory diseases (132). In addition, Huang et al. demonstrated that SCFA played a protective role in obesity-related asthma by modulating immune responses. SCFA levels were reduced in individuals with obesity, contributing to increased activation of pro-inflammatory Th17 cells and elevated production of IL-17. The study suggested that restoring SCFA levels might help regulate immune cell activity and reduce airway inflammation in obesity-related asthma (133). Interestingly, Sun et al. showed that curcumin increased the SCFA levels in mice with myasthenia gravis which played a crucial role in modulating gut immunity. Indeed, higher SCFA levels promoted healthier gut microbiota and improved the immune balance by reducing pro-inflammatory Th17 cells and enhancing anti-inflammatory Tregs activity. This shift in the Th17/Treg balance helped mitigate inflammation and protect against autoimmune responses, highlighting the essential role of SCFAs in maintaining gut immune homeostasis (134). Zhao et al. found that Entomopathogenic fungi-derived metabolite Ento-A mitigated the DSS-induced colitis in mice by enhancing the production of SCFAs which were crucial for modulating immune responses. The increase in SCFA levels inhibited the pro-inflammatory Th17 signaling pathway, resulting in decreased gut inflammation (135).

Interestingly, growing evidence supports that SCFAs have both tissue- and disease-specific effects as described below.

### Tissue-specific effects of SCFAs

In the liver, butyrate and propionate are known to promote an anti-inflammatory Kupffer cell phenotype, reduce steatosis and induce Tregs, which protect against non-alcoholic steatohepatitis (NASH). The key mechanisms involve GPR41/GPR43 signaling and the regulation of enzymes of the lipid metabolism pathway. Similarly, in colonic and gut tissues, butyrate, propionate, and acetate promote the differentiation of Foxp3+ Tregs, inhibit the expression of proinflammatory cytokines such as IL-6 and IL-17, reduce Th17 cell expansion, and maintain the integrity of the epithelial barrier. The key mechanisms involve GPR43/GPR109A activation, HDAC inhibition, and HIF-1α stabilization. In the lung tissue, propionate and acetate promote the tolerogenic DCs and modulate Tregs and ILC2 populations, which protect against allergic asthma. The key mechanisms involve GPR41/GPR43 signaling and HDAC inhibition. In the central nervous system (CNS), via the gut-brain barrier, butyrate and propionate influence microglial maturation and activation and regulate neuroinflammation. The key mechanism involves the regulation of CNS-resident immune cells. Regarding systemic effects (blood and spleen), butyrate and propionate support Treg proliferation and suppress the systemic levels of proinflammatory TNF-α and IL-6. There is reduced activation of autoimmune T- and B-cells (107). The key mechanism involves epigenetic modulation of immune cell precursors (**Figure 2**).

### Disease-specific effects of SCFAs

Regarding autoimmune diseases, in IBD, butyrate supports the proliferation of gut Tregs, suppresses the IL-17/Th17 axis, and improves the integrity of the gut barrier (136). The key mechanisms involve GPR109A signaling and HDAC inhibition (**Figure 2**), which alleviates colonic inflammation and improves repair of the gut mucosa. In rheumatoid arthritis, SCFAs suppress joint inflammation by altering Th17/Treg balance and through SCFA-dependent modulation of systemic cytokines, leading to reduced autoantibody formation and less joint inflammation. In type 1 diabetes (T1D), butyrate and acetate suppress islet-specific T-cells and promote tolerogenic DCs and macrophages (137). The SCFA-associated epigenetic modulations lead to delayed T1D onset and reduced disease severity. In multiple sclerosis (MS), butyrate and propionate promote the systemic and CNS-resident Tregs and reduce microglial activation. This mechanism involves SCFA-associated effects on the gut-brain barrier (138).

Regarding infectious diseases, in bacterial gut infections, SCFAs reduce the colonic pH, which inhibits the growth of pathogens. Also, mucosal IgA levels are upregulated, conferring local immunity and antimicrobial peptide induction. These changes lead to reduced gut bacterial colonization and inflammation (51). In viral infections, such as HIV and SARS-CoV-2, SCFAs restore a balance between inflammation and tolerance, supporting local immunity and gut barrier function. The key mechanism involves microbiome-dependent effects on immune checkpoints, leading to reduced leaky gut and less viral spread (139). In lung infections, SCFAs improved lung inflammation, and the mechanisms involved GPR41/GPR43 interactions, and increased Tregs and TGF-β levels (140). These changes led to reduced lung inflammation and increased viral clearance. In sepsis, SCFAs suppressed systemic inflammatory cytokines and reduced monocyte/macrophage activation (141). This epigenetic reprogramming of innate immune responses resulted in reduced lethality and improved tissue homeostasis and immune tuning.

However, it is noteworthy that butyrate treatment during Treg differentiation can result in the induction of transcription factor T-bet, which suppresses Foxp3 expression. This indicates a reduction in Treg-mediated immunosuppression. Also, butyrate has been found to upregulate expression of proinflammatory cytokines, such as IFN-γ, suggesting a proinflammatory Th1 shift and reduction in Treg responses (142) Interestingly, in these contexts, butyrate has been shown to specifically inhibit the HDAC3 levels, which suggests a potential mechanism for its gene regulatory effects. Overall, the role of HDAC3 in butyrate-Treg interaction is still underexplored (**Figure 2**). Probably, the overall effect of butyrate on Tregs results from its interplay with different immune cell types and crosstalk with complex signaling pathways, rather than a single direct effect.

While SCFAs are well-studied, several other classes of microbial metabolites are also important for regulating immune tolerance. These metabolites include the following: (i) Trp metabolites (143): Gut microbiota (*Lactobacilli*, *Clostridia*, other commensals) metabolize dietary Trp into indole derivatives, including indole-3-aldehyde, indole-3-acetic acid, indole-3-propionic acid, and kynurenine. These metabolites modulate host immunity via activating AhR signaling and other pathways (IL-22, NRF2) in Tregs; (ii) Secondary bile acids (144): Certain gut microbiota (*Clostridia*spp., *Bacteroides*) modify the host-derived primary bile acids, which have immunomodulatory properties. Such key metabolites include deoxycholic acid and lithocholic acid, and its derivatives, which inhibit Th17 differentiation and promote Foxp3+ Treg responses by RORγt inhibition. These cues suppress Th17 cells, and promote Treg stability and immune tolerance; (iii) Polyamines’ expression (145): Microbial amino acid (especially arginine) metabolism produces polyamines, such as spermine, spermidine, and putrescine, that promote Treg differentiation and stability, reduce inflammation, and enhance epithelial barrier integrity; (iv) Expression of polysaccharide A and exopolysaccharides (146): Certain bacteria produce complex carbohydrates, such as polysaccharide A (from *Bacteroides fragilis*) and exopolysaccharides (from *Lactobacillus rhamnosus*), that promote tolerogenic DCs phenotypes, IL-10 production, and Foxp3+ Treg expansion; and (v) Microbial vitamins and co-factors (147): Certain gut microbes produce vitamins and co-factors, such as vitamin B2/riboflavin (produced by *Bifidobacterium*and *Lactobacilli*), vitamin B9/folate (produced by many commensals in the gut), and vitamin K2/menaquinone (produced by *Bacteroides*and *Eubacterium*), that promote Treg survival and function, activate mucosal-associate invariant T (MAIT) cells, and support mucosal immunity and integrity.

### Mucosal barrier function

Another important mechanism is the interaction between gut microbiota and intestinal epithelial cells. The microbiota can affect the integrity and function of the gut epithelial barrier which, in turn, influences immune responses. It was demonstrated that certain gut bacteria could produce factors that strengthened the epithelial barrier, reducing the likelihood of inflammatory responses triggered by the leakage of gut contents into the systemic circulation. This barrier function was crucial for preventing the development of immune-mediated diseases and maintaining overall immune homeostasis (44).

Another key mechanism by which the gut microbiota helps maintain the mucosal barrier integrity is the regulation of tight junction (TJ) proteins, such as occludin, zonula occludens (ZO)-1, and claudins (113). TJ proteins are the complexes that seal the space between epithelial cells, giving rise to a selectively permeable barrier that regulates the passage of biomolecules and microbes from the gut lumen into the lining tissues. Occludin is an integral membrane protein that regulates barrier permeability. ZO-1 is a scaffolding protein that links transmembrane proteins with the actin cytoskeleton. Claudins are the proteins that control paracellular permeability. Disruption of these TJ proteins leads to loss of selective permeability function, a condition called leaky gut, which is associated with inflammatory, metabolic, and autoimmune disorders. By enhancing the expression and function of TJ proteins, certain gut microbiota strengthen the mucosal barrier, e.g., a mucin-degrading commensal bacterium, *Akkermansiamuciniphila*, found in the colonic mucous layer, invigorates the integrity of the gut barrier by upregulating the expression of TJ proteins (occluding, ZO-1) on epithelial cells and by increasing the mucin (MUC2) production, which leads to lower gut permeability and systemic inflammation. In both mice (148) and humans (149), *Akkermansia*administration improved metabolic profiles, controlled endotoxemia, and alleviated IBD symptoms. Microbiota regulate the TJs via the following key mechanisms (150): (i) SCFAs (e.g., butyrate), produced by gut microbiota like *Akkermansia*and *Firmicutes*, upregulate the expression of occluding/ZO-1 as well as increase the production of anti-inflammatory cytokine IL-10; (ii) Microbial components, such as LPS and peptidoglycan, induce the controlled activation of TLR (TLR4/TLR2) signaling and modulate TJ proteins expression without triggering inflammation; (iii) Gut microbiota metabolites enhance the expression of certain transcription factors, such as NF-κB and HIF-1α, leading to regulation of TJ protein expression; and (iv) By mechanism of competitive inhibition and through the expression of antimicrobial peptide which protects from pathogenic damage to the gut barrier. Since microbiota-regulated TJ proteins (occluding/ZO-1) play a role as essential gatekeepers of gut barrier function and protect against pathogen invasion, inflammation, and systemic disease, restoring a healthy microbiome and/or targeting these key pathways could offer therapeutic potential in gut and systemic disorders (151). The relevance of microbiota-regulated TJ proteins to protective responses in obesity, diabetes, IBD, cancer, and neurodegenerative disorders has been documented.

Recent findings also suggest that gut microbiota affects immune tolerance through interactions with the GALT. GALT plays a critical role in distinguishing between pathogenic and non-pathogenic antigens, and the microbiota can influence this process by modulating the local immune environment. To this effect, certain bacteria may promote the development of tolerogenic DCs which help in inducing tolerance by presenting antigens in a way that minimizes inflammatory responses (109). Future research needs to focus on identifying the specific microbial profiles that are associated with dysbiosis and developing novel strategies to restore the balanced microbiota to enhance immune tolerance and prevent disease development.

## Role of microbial tryptophan metabolites in programmed death (PD)-1/PD-L1 tolerance

Microbial Trp metabolites play a critical role in immune tolerance, including the PD-1/PD-L1 pathway (152–154). An overview of this immunomodulatory crosstalk is presented below.

### Trp metabolism and microbiome

Trp is an essential amino acid that is metabolized in several pathways as follows. (i) Microbial/Indole pathway: Gut microbes convert Trp into indole and other derivatives, such as indole-3-aldehyde, indole-3-acetic acid, and indole-3-propionic acid; (ii) Kynurenine pathway: Occurs mainly in host cells via indoleamine 2,3-dioxygenase or tryptophan 2,3-dioxygenase, producing immunosuppressive metabolites, such as kynurenine; and (iii) Serotonin pathway: Serotonin and melatonin are produced. Notably, microbial metabolites, particularly indoles, are potent immune modulators.

### PD-1/PD-L1 pathway

The PD-1/PD-L1 pathway is a crucial immune checkpoint mechanism that plays a pivotal role in immune regulation and the maintenance of self-tolerance. PD-1 receptor is expressed on activated T-cells, B-cells, and some myeloid cells. PD-1 acts as an inhibitory receptor to suppress immune responses. Its cognate ligands, PD-L1 and PD-L2, are expressed on APCs and tumor cells. However, PD-L1 is most widely studied in the context of tumor immunology. PD-1/PD-L1 engagement generates an inhibitory signal that leads to T-cell inhibition, tolerance, and immune evasion. The outcome is reduced T-cell proliferation, diminished cytokine expression, and impaired cytotoxic responses. Hence, PD-1/PD-L1 pathway activation limits tissue damage during inflammation and also maintains immune tolerance by preventing autoimmune overactive T-cells. Not surprisingly, PD-1/PD-L1 pathway is a key immune checkpoint that tumors exploit for immune evasion, and targeting this pathway has revolutionized cancer immunotherapy.

### Effect of microbial Trp metabolites on PD-1/PD-L1 pathway

Microbial tryptophan metabolites can induce PD-1/PD-L1-mediated tolerance through the following mechanisms: (i) Microbial metabolites, such as kynurenine, can downmodulate T-cell function as well as promote exhausted T-cell phenotypes with increased PD-1 receptor expression; (ii) Several microbial Trp metabolites, including indole-3-aldehyde, can act as AhR ligands and activate AhR-mediated signaling in immune cells, resulting in increased Treg differentiation, PD-1 receptor induction on T-cells, and enhanced PD-L1 expression on macrophages and DCs. These changes promote an immunosuppressive environment and induce immune tolerance; and (iii) Microbial indoles trigger AhR-mediated signaling, which leads to increased production of anti-inflammatory cytokines, such as TGF-β and IL-10. These anti-inflammatory cytokines further potentiate PD-1/PD-L1-mediated T-cell inhibition and immune tolerance (72).

It is notable that in the gut, microbial Trp metabolites promote the PD-1/PD-L1 pathways and play a significant role in maintaining tolerance to commensal bacteria. In cancer, on the other hand, these metabolites enhance the PD-L1 expression in the tumor microenvironment, contributing to immune evasion (154). In conclusion, microbial Trp metabolites, via AhR signaling and other pathways, can induce PD-1/PD-L1-mediated immune tolerance and reinforce mucosal immunity; however, these microbial metabolites also contribute to tumor immune evasion.

## Emerging concepts and promising new microbiome-based targets

### Microbiota-derived extracellular vesicles

MEVs are nano-scale membrane-bound particles or vesicles secreted by microorganisms in the human microbiome, in areas such as the gut, skin, and oral cavity. The MEVs deliver a variety of molecular cargo, including nucleic acids, lipids, proteins, and small metabolites, and play critical roles in communication, host physiology, immune function, and disease progression (155). Based on microorganism types, MEVs are classified as follows: (i) Outer membrane vesicles (OMV; 20–250 nm size) produced by outward budding of the outer membrane of Gram-negative bacteria following stress, quorum sensing, or selective pressure of antibiotics. OMVs contain the outer membrane proteins, LPS, nucleic acids (DNA/RNA), and virulence factors (156); (ii) The membrane vesicles (MVs; 20–150 nm size) of Gram-positive bacteria, produced by cell wall remodeling or regulated autolysis, allowing passaging of bioactive molecules across the thick peptidoglycan layer of the bacterial cell wall (157). These MVs contain the lipoteichoic acid, peptidoglycan fragments, and cytoplasmic proteins; and (iii) EVs produced by Fungi and Archaea, which contain different bioactive molecules (158). Biogenesis mechanisms of MEVs include the regulated active secretion or the passive release following stress or cell lysis.

### Cargo composition

The cargo composition of MEVs is highly diverse. The bioactive molecules present mostly comprise nucleic acids (DNA, mRNA, and small RNAs, including miRNA-like molecules and sRNAs), proteins (enzymes, adhesion molecules, and toxins), lipids (LPS, lipoteichoic acid, and phospholipids), and metabolites (SCFAs and signaling molecules) (155). This bioactive cargo is selectively packaged and represents the physiological state of the parent microbiota.

### Functions of MEVs

Overall, MEVs orchestrate the following critical functions: (i) Inter-microbial communication through mechanisms such as horizontal gene transfer, biofilm formation, and anti-bacterial activities via bacteriocins; (ii) Host-microbiota interactions, such as barrier function affecting epithelial permeability and tight junctions, immune modulation by activating or suppressing TLR-mediated immune responses, and neuroimmune signaling by influencing immune and neural pathways in the gut-brain axis; (iii) Pathogenesis through delivering virulence factors to host cells, by suppressing host defenses, and contributing to chronic infections such as IBD and periodontitis; and (iv) Role in host health by maintaining gut membrane integrity or barrier function, stimulation of Tregs, and bolstering mucosal immunity (159–161).

### Roles in disease and health

In IBD, proinflammatory MEVs from pathogenic bacteria exacerbate gut inflammation (162). In neurodegenerative disorders, MEVs can deliver proinflammatory signals across the gut-brain axis (163). In cancer, certain MEVs can modulate the tumor microenvironment and promote metastasis (164). In infections, pathogenic MEVs can deliver toxins and compromise immunity (165). In metabolic diseases, MEVs have been found to play a role in adipose inflammation and insulin resistance (166).

Regarding the diagnostic and therapeutic potentials of MEVs, their profiling in blood and feces may serve as biomarkers of microbiota and disease states. Interestingly, OMVs are being assessed for vaccination potential, such as *Neisseria meningitidis*based OMV vaccine (167). In the field of drug delivery research, engineered MEVs are being used for targeted delivery (160). Probiotics-derived EVs are being explored for their therapeutic effects without requiring the delivery of live microbiota (168). Notably, current challenges guiding future research directions include the standardization methods for isolation and analysis of MEVs, understanding the mechanisms of MEV biogenesis and cargo selection, and optimization for their clinical translation involving safety, scalability, and MEV delivery studies.

## Bacteriophage-immune crosstalk as underexplored targets

Bacteriophages, or phages, are viruses that infect bacteria, and the emerging research shows that the phages also interact directly or indirectly with the host immune system, in a process called phage-immune crosstalk. It is noteworthy that such interactions have largely remained underexplored, despite critical significance for immunomodulation, therapeutic development, and microbiome engineering. The classical view supports that phages indirectly affect human health by shaping the microbiome through regulating bacterial populations. While, the emerging view supports that phages can also act as immune stimuli or immunomodulators by interacting directly with host immune cells and play a role in health and disease (168, 169).

### Mechanisms of phage-immune crosstalk

In direct interaction mechanisms, phages can be recognized and internalized by host innate immune cells, including monocytes/macrophages and dendritic cells. The pattern recognition receptors (PRRs) present on these cells get selectively activated by various phage components as follows: (i) TLR3/7 recognize dsRNA/ssRNA; (ii) TLR9 recognizes unmethylated CpG DNA; and (iii) the cGAS-STING pathway is activated by cytosolic DNA sensor. Such interactions lead to the induction of various cytokines, type I interferon (IFN-α/β), and modulation of antigen presentation. Phages impact human health by indirectly altering bacterial populations and shaping the microbiome as follows: (i) By releasing LPS, peptidoglycan, or toxins, which modulate immunogenicity; (ii) By altering metabolite profiles, which influence immune responses via Tregs and Th17 cell responses; and (iii) By altering immune accessibility through biofilm structural changes. However, phages from the gut virome may also translocate across the compromised gut barrier during disease conditions, such as IBD and HIV. In the circulation, phages may interact with critical systemic immune components, including the phagocytes, immunoglobulins (IgG/IgA), and the complement system (170, 171).

### Emerging evidence from pre-clinical and clinical studies

Using *in vitro*macrophage model, it was found that the exposure to phages leads to activation of inflammatory or tolerogenic responses, which depended on phage types (172). In mouse model studies, it was shown that oral administration of phages modulated the production of cytokines, including IFN-γ and IL-10, protecting against colitis (173). Moreover, fecal virome transplantation (FVT) was found to restore immune and metabolic balance, both in antibiotic-treated and germ-free mice (174). In humans, the alteration of gut virome composition was related to immune changes in diabetes, IBD, and Parkinson’s disease (175).

### Therapeutic potential

Phages have vast potential for diverse uses, summarized as follows: (i) Phages or their components, such as CpG DNA, can act as natural adjuvants to boost immunity or activate DCs in cancer immunotherapy; (ii) Tumor-associated phages may influence tumor immune evasion, modulating T-cell infiltration and responses of tumor-associated macrophages; and (iii) Phages also act as delivery vehicles for immunostimulatory genes. Notably, phages with anti-TNF immunosuppressive potential can be used as a therapeutic tool for inflammatory and autoimmune diseases. Similarly, alterations in the gut virome in colitis and multiple sclerosis reduced inflammation and disease severity (176, 177).

### Major challenges and future directions

Firstly, phage biodistribution and kinetics *in vivo*have yet to be fully characterized. Secondly, the immune response varies due to phage diversity and specificity, phage preparations, and host genetic variation, such as TLR polymorphisms. Thirdly, cross-reactive antibodies may neutralize therapeutic phages.

As non-living entities, phage-derived nanoparticles are gaining attention as programmable immune modulators. Synthetically engineered phages are becoming a useful tool to deliver RNAs or immunomodulatory peptides. In precision medicine, virome-targeted microbiome therapies are being used as a tool for gut virome tailoring. Using systems biology approaches, phage-immune networks are being mapped to understand, predict, and model interactions.

In conclusion, phage-immune crosstalk represents an emerging frontier in immuno-microbiome research. Although relatively still underexplored, it holds vast potential for developing new immuno-therapeutics and phage-based interventions. Continued research to gain an in-depth understanding of these interactions could pave the way for breakthrough treatments for metabolic, inflammatory, infectious, and neoplastic diseases (178, 179).

## Metabolite-immune checkpoint axis: a new conceptual framework

As an emerging conceptual framework, MICA links host and microbial metabolites with the regulation of immune checkpoints, specifically in disease conditions such as inflammation, cancer, and immune tolerance. It is now being recognized as a promising interdisciplinary concept in microbiome research, immunometabolism, and immunotherapy.

MICA represents the regulatory interactions between metabolites (from host cells, microbiota, or diet) and immune checkpoint pathways (PD-1/PD-L1, LAG-3, CTLA-4, and TIM-3). These regulatory pathways play a critical role in controlling immune activation and preventing autoimmunity. Emerging evidence shows that metabolic signals, especially from microbiota, modulate these checkpoint pathways (180), indicating a promising yet underexplored avenue of immunoregulation research. Butyrate, a gut microbiota SCFA, supports Treg development and reduces PD-1 expression (181). Lactate, expressed by immune and tumor cells, upregulates PD-L1 expression in macrophages (182). Succinate and fumarate, which are TCA cycle intermediates, also upregulate PD-L1 expression by inducing the expression of HIF-1α (183). Kynurenine, a metabolite of tryptophan catabolism, stimulates AhR and induces the expression of CTLA-4 and PD-1 (184). Adenosine, expressed during cell stress, binds to A2A receptor and enhances PD-1 expression, supporting immunosuppression (72). Trimethylamine N-oxide (TMAO), a microbial metabolite, induces an inflammatory response via NF-κB upregulation (185). Inosine, a metabolite produced by gut microbiota, induces T-cell activation and supports inflammation (186).

As an emerging conceptual framework, MICA interlinks the critical pathways of immune tolerance, metabolism, and microbiota-host co-metabolism under one integrated framework. This approach may lead to novel therapeutic horizons by allowing for dual-target strategies in which metabolic pathway modulators may be combined with immune checkpoint inhibitors to gain a desirable outcome. A few of such innovative therapeutic approaches may involve butyrate + anti-PD1 or IDO inhibitors + CTLA-4 blockade strategies. Moreover, individual metabolomic profiles may guide such dual therapeutic strategies for further refinement of targeted outcomes. Besides, circulating or fecal metabolites may serve as non-invasive biomarkers for immune checkpoint modulation and the therapeutic responses induced.

Indeed, mapping metabolite-checkpoint interactions using multi-omics approaches, e.g., metabolomics combined with immunophenotyping, needs to be further focused. Microbiota engineering may yield target metabolites that modulate immune checkpoints to the desired outcome. The AI-based modeling can help predict MICA signatures in various disease contexts. More importantly, clinical trials of metabolite+immune checkpoint regulators-based combined therapies remain a highly desirable direction for future research. In conclusion, as a promising conceptual framework, MICA reframes how we understand immune checkpoints, not as static receptors but as a dynamic framework influenced by the metabolic milieu. Exploring this potential axis may revolutionize immunotherapy and precision medicine as well as our understanding of immune tolerance and dysfunction.

## Gut microbiota-based therapy

Numerous research efforts across the globe have concentrated on the profound influence of gut microbiota on human health and disease. While there is still a considerable amount of fundamental work required, the primal connection between human well-being and gut health has been recognized a long time ago as evidenced by Hippocrates’ assertion in 400 B.C. stating that “Death sits in the bowels” (187).

The gut microbiota, a complex community made up of trillions of microorganisms residing in the human gastrointestinal (GI) tract, plays a pivotal role in health and disease. The concept of gut microbiota-based therapy has gained significant traction as a promising approach to treat a variety of morbid conditions, ranging from GI tract disorders to metabolic and neurodegenerative diseases.

The latest developments in gut microbiota-based therapies, focusing on probiotic interventions (188), fecal microbiota transplantation (FMT), and prebiotic supplementation are summarized below.

### Probiotics and their therapeutic potential

Probiotics are the live microorganisms that confer health benefits when administered in adequate amounts and have been extensively studied for their potential to modulate gut microbiota. Recent randomized controlled trials have demonstrated that specific strains of *Lactobacillus*and *Bifidobacterium*can alleviate the symptoms of IBS and IBD by restoring microbial balance and reducing inflammation (189). An elegant study demonstrated that multi-strain probiotic formulations were more effective than single-strain probiotics in improving gut barrier function and modulating the immune response. However, the effectiveness of probiotics could be highly strain-specific and influenced by the host’s baseline microbiota, necessitating personalized approaches for optimal outcomes s (190). This variability in efficacy across clinical trials has been a major limitation in translating findings into standardized treatment protocols. In corroboration with these findings, Bashir et al. suggested that replenishing *Lactobacillus plantarum*in aged mice could restore the tolerogenic function of DCs by modulating inflammatory and metabolic pathways. As a therapeutic option, *Lactobacillus plantarum*could potentially be used to treat age-related immune dysfunction and disorders associated with gut dysbiosis (81).

### Fecal microbiota transplantation

FMT has traditionally been used to treat recurrent *Clostridioidesdifficile*infections with remarkable success. However, recent studies have expanded its potential applications to include conditions such as ulcerative colitis and metabolic syndrome (191) as well as neurological disorders like Parkinson’s disease, and physiological conditions such as autism, schizophrenia, and Rett syndrome (192). Notably, Tian et al. demonstrated that FMT could induce remission in patients with refractory ulcerative colitis, with a success rate comparable to biologic therapies (193). Furthermore, emerging data suggest that FMT may alter the gut-brain axis, offering a novel therapeutic axis for managing neuropsychiatric conditions, such as Alzheimer’s disease (194). Despite these promising results, standardization of FMT procedures and long-term safety data remain elusive. Variability in donor stool composition, delivery method (e.g., capsule vs. colonoscopy), and regulatory oversight further complicate clinical outcomes. The FDA currently limits non- C. difficile FMT use to Investigational New Drug (IND) protocols, as live biotherapeutic products (LBPs) are regulated under biologics frameworks.

### Prebiotics and dietary interventions

Prebiotics are the non-digestible food components that promote the growth of beneficial gut bacteria and are considered another cornerstone of microbiota-based therapy. Recently, specific prebiotics, such as inulin and fructooligosaccharides (FOS), have been identified as the substances that selectively enhance the growth of beneficial microbial taxa, including *Bifidobacteria*(195). These prebiotics have shown potential in reducing the risk of metabolic diseases, such as T2D, by modulating the gut microbiota and improving glucose metabolism (196). Additionally, dietary interventions that include a high intake of fiber-rich foods are being increasingly recognized as crucial for maintaining gut health (197). The synergistic effects of combining prebiotics with probiotics, known as synbiotics, are currently under investigation and may represent a more effective strategy for modulating the gut microbiome (198). To better understand these interventions’ clinical utility, we summarize selected trials evaluating probiotics, FMT, synbiotics, and SCFA-based therapies in **Table 2** below. These studies illustrate promising outcomes but are limited by small sample sizes, endpoint heterogeneity, and lack of mechanistic depth.

**Table 2:** Summary of selected clinical trials evaluating gut microbiota-based therapies.

Trial identifier	Intervention	Condition	Sample size	Primary outcome	Key findings	Limitations	References
NCT02254811	FMT (oral capsules vs. colonoscopy)	Recurrent *Clostridioides difficile*Infection	116	Clinical cure at 8 weeks (infection resolution)	Capsule-delivered FMT non-inferior to colonoscopy; cure rates ~90%	Large-scale CDI not IBD; lacks long-term immunologic/microbiome endpoints	(199)
NCT04089042	*Lactobacillus plantarum*PS128	Parkinson’s Disease	60	Motor symptom improvement	Reduced UPDRS scores; gut-brain axis modulation	Small sample size; no placebo control	(200)
NCT04388577	Synbiotic (*B. longum*+ Inulin)	Metabolic Syndrome	80	Fasting glucose reduction	Improved insulin sensitivity	Short duration (12 weeks); no microbiota sequencing	(201)
NCT03699972	FMT (colonoscopy)	Autism Spectrum Disorder	18	GI and behavioral symptom improvement	Improvements sustained at 8 weeks	Open-label; small N	(202)
NCT03819242	SCFA Enemas (Butyrate)	Ulcerative Colitis	40	Mucosal healing	Increased Tregs; reduced inflammation	Local delivery; unclear long-term effect	(203)

On the note, the recent research has highlighted several innovative gut microbiota-based therapies. In this regard, postbiotics, such as SCFAs, have shown the potential therapeutic applications in various inflammatory diseases (204). Bacteriophage therapy involves using the viruses that target specific pathogenic bacteria in the gut, providing a way to modulate microbiota composition and treat conditions like *Clostridium difficile*infections without disturbing the overall gut microbiome (205). Additionally, engineered microbiota, achieved through genetic modifications of gut bacteria, holds promise for treating GI tract diseases and metabolic disorders by enabling the production of therapeutic compounds directly within the gut (206). These emerging therapies represent the cutting-edge advancements in gut microbiota research and are being explored for their broad-scale therapeutic potential.

## Current advancements in personalized therapies utilizing the microbiota to regulate immune tolerance

### Personalized FMT

- Next-generation FMT, utilizing donor stools matched to the recipient’s immune or microbial profiles (207, 208).
- Trials using FMT to enrich Treg-promoting bacteria.

Examples: *Faecalibacteriumprausnitzii*, *Clostridia*clusters, being used in several diseases, such as Type 1 diabetes, and ulcerative colitis (209, 210).

### Defined microbial consortia

Synthetic consortia, comprising of well-defined commensal strains, have been designed to address the following uses (211–213):

- To enhance Treg induction.
- To mitigate Th1/Th17 pro-inflammatory responses.

Examples: (i) Clostridia-based consortia for immune tolerance in IBD (VE-202), Vedanta Bioscience (214); and (ii) Oral live bacterial therapeutics (SER-155, SER-301), Seres Therapeutics (www.serestherapeutics.com).

### Microbiome metabolite targeting

Personalized approaches using host genetics and microbiota composition to design or select optimal metabolite modulators, including therapies that mimic or potentiate tolerogenic microbial metabolites, such as (215, 216):

i. Indole derivatives (Metabolites of Trp metabolism, serving as AhR ligands).
ii. Secondary bile acids (oxo-LCA, iso-LCA).
iii. Polyamines and vitamin B9.

### Microbiome profiling targeting immunotherapy responses

Using personalized immunotherapies for integrating microbiome signatures to (217):

- Identify microbiota-related immune tolerance defects.
- Predict responses to immune checkpoint inhibitors (ICIs).

Examples: Microbiome modulations using diet, probiotics, and FMT, in combination with immunotherapy to enhance efficacy and immune tolerance.

### Using pre- and post-biotics

- Tailored prebiotics, such as specific fibers or polyphenols, are being used to selectively enrich Treg-promoting bacteria.
- Postbiotics, such as purified bacterial metabolites (e.g., butyrate, from *Bacteroides fragilis*).

Examples: (i) IBD; (ii) Neuroimmune disorders; and (iii) food allergies (218, 219).

## Conclusions and future perspectives

The gut microbiota holds great promise as a modifiable target for enhancing immune tolerance and treating a variety of disease conditions. The gut microbiota-based therapeutic interventions such as probiotics, prebiotics, FMT, bacteriophage therapy, and engineered microbiota have, indeed, shown success in treating diseases and improving human health. However, several challenges still remain to be duly addressed. The microbiome complexity and variability in the individual microbiomes, influenced by other potentially interactive factors such as genetics, diet, age, health status, and environment, complicate the standardization of the therapeutic approaches (220). Personalized microbiota-based therapies represent a promising frontier in immune tolerance modulation, leveraging advances in metabolomics, microbial profiling, and synthetic biology. Such approaches offer new hope for treating chronic inflammatory disorders, autoimmune diseases, and allergies with profound precision and minimal side effects. The gut microbiota dynamically shifts in response to lifestyle changes or treatments and such microbiome shifts make predicting and maintaining the effectiveness of personalized therapies a consistent challenge to deal with. This variability makes it hard to develop a ‘one-size-fits-all’ kind of therapy, while the tailored interventions necessitate a critical and deep understanding of microbiome profiles and their interaction with genetics, age, drugs, and environment. Indeed, to personalize treatments effectively, we will need to identify more reliable biomarkers or microbial signatures that can successfully predict the outcome of complex interactions between the microbiomes and the host physiology. For therapies like FMT, prebiotics/probiotics, or bacteriophage-based treatments, it is imperative to comprehend which microbiota compositions or specific strains would be crucial for different conditions. In the absence of clear biomarkers, it would be arduous to know whether a particular treatment can provide long lasting benefits. Moreover, the long-term effects of microbiome modulations are still not fully understood as most microbiota-based therapies are relatively new. The plausible risks of unintended consequences, such as dysbiosis or overgrowth of harmful microbes, require more intensive investigations. Importantly, the use of probiotics or prebiotics over extended periods of time as well as adoption of more invasive approaches like FMT or engineered microbiota can likely cause some side effects, including imbalances in the gut microbiome. Besides, the long-term effects on host immune system, metabolism, and other vital systems are still not fully understood.

It is also notable that the formulations, doses, and quality control of microbiome-based interventions vary widely, and the established standards are largely non-existent. This may be attributed to variations in the potency and efficacy of probiotic strains, as well as the need for standardized protocols in fecal microbiota transplantation (FMT) procedures. Understandably, the regulatory environment for these therapies is still evolving and there is uncertainty about how to classify and regulate these procedures. FMT approaches also involve critical ethical concerns, such as donor screening and potential risks of transmitting infectious diseases or unintended microbiota. Similarly, other therapies involving engineered microbiota or bacteriophages raise concerns about the likelihood of causing adverse side effects or unintended microbial interactions. Given that the gut microbiota consists of trillions of microorganisms, such interactions may become highly complex, making it challenging to accurately predict the outcome of specific interventions. Thus, identifying the subgroups that will benefit the most from such therapies remains a major concern in the field. Future research should aim to create more successful personalized or individually customized gut microbiota-based therapies. Further technological and high-throughput methodological advances in microbiome sequencing, bioinformatics, and synthetic biology are expected to lead to more effective probiotics and potentially tailored FMTs across diverse human populations. At present, clinicians are challenged with difficulties regarding the translation of microbiome research into personalized treatments due to the inherent complexity of microbiome data. The integration of microbiome profiling with clinical care can be improved by using new tools, further training, and more integrated workflows.

Indeed, large-scale clinical trials will be essential to test and validate the efficacy and safety of these gut microbiota-based therapies for diverse population groups. Such advancements in knowledge will allow for a deeper understanding of the therapeutic responses as well as better prediction of the clinical outcome. As a word of caution, the current prohibitive costs involved in using FMT or engineered microbiota may limit their access to many individuals, especially across low-resource settings and, therefore, the future research also needs to identify more cost-effective approaches for large-scale adoption of microbiome-based therapies. Besides, MEVs represent an emerging frontier in microbiome research, offering promising insights into host-microbiota interactions and as potential tools/targets for novel diagnostics/therapeutics. MEV-based interventions have vast potential in precision medicine. Overall, as research progresses, personalized microbiome-based therapies will most likely become a cornerstone of precision medicine, aiming at restoring or potentiating immune tolerance in ways that are safer and more effective.
